# Trends in mortality and comorbidities in hemodialysis patients between 2012 and 2017 in an East-European Country: a retrospective study

**DOI:** 10.1007/s11255-023-03549-6

**Published:** 2023-03-14

**Authors:** Lazar Chisavu, Adelina Mihaescu, Flaviu Bob, Alexandru Motofelea, Oana Schiller, Luciana Marc, Razvan Dragota-Pascota, Flavia Chisavu, Adalbert Schiller

**Affiliations:** 1 University of Medicine and Pharmacy “Victor Babes” Timisoara, Timisoara, Romania; 2 Avitum BBraun Dialysis Centre, Timisoara, Romania; 3 Emergency County Hospital “Pius Brinzeu” Timisoara, Timisoara, Romania; 4 Emergency City Hospital of Timisoara, Timisoara, Romania; 5 Emergency Hospital for Children “Louis Turcanu” Timisoara, Timisoara, Romania

**Keywords:** Hemodialysis, Mortality, Cardiovascular disease, Comorbidities, Cardiovascular death, Survival

## Abstract

**Purpose:**

The aim of this study was to evidence trends and changes in mortality, comorbid conditions, prognosis, and causes of death after 5 years of continuous evolution of hemodialysis (HD) patients in Romania.

**Methods:**

We included two cohorts of stable HD patients (901 from 2012 and 1396 from 2017). Both cohorts were followed up for 1 year. The 5-year survivors of the 2012 cohort were identified in 2017 and their data changes were assessed.

**Results:**

The 2017 patients were older, with longer time on dialysis, higher serum creatinine and urea levels, and required higher ultrafiltration volume per dialysis. They also had lower hemoglobin, lower C-reactive protein, higher albumin, higher calcium bicarbonate, and higher parathyroidectomy prevalence. The 2017 cohort presented with lower average dialysis flow, less administration of iron sucrose, had more catheters, lower hepatitis C prevalence, higher diabetes mellitus prevalence, higher heart valve calcifications, higher heart rate disorders, higher prevalence of left ventricular hypertrophy, and lower ejection fraction. Cardiovascular disease was the main cause of death in both years (50% in 2012 and 45.6% in 2017), followed by sepsis and cancer. The mortality was higher in 2017 compared to 2012 (14.1 vs 6.6%). The 5-year mortality was 37.2% with an average of 7.44%/year. The risk of death increased with age, higher C-reactive protein, higher phosphate, lower hemoglobin, and lower albumin.

**Conclusion:**

Cardiovascular disease remains the main causes of death in HD-treated patients but with decreasing trend. Developing regional therapeutic strategies for quality care with early intervention will most likely improve mortality.

## Introduction

The advances of chronic kidney disease (CKD), end-stage kidney disease (ESKD), and their comorbidities/complications treatment over the last decades increased the survival of patients on hemodialysis therapy. The mortality, however, remained high, being 10 to 30-fold, higher than in the general population [1]. The major renal registries, European Renal Association-European Dialysis and Transplant Association (ERA-EDTA) and United States Renal Data System (USRDS), reported the annual mortality, in 2019, as 14.6% in Europe [2] and 15.6% in the United States, respectively [3].

Cardiovascular disease (CVD) remains the first cause of mortality in hemodialysis (HD)-treated patients. In 2019, 55% of deaths were attributable to CVD according to USRDS Annual Report (i.e., arrhythmia/cardiac arrest, acute myocardial infarction, congestive heart failure, and stroke) [3]. The prevalence of cardiovascular disease is very high in ESKD patients with up to two-third of them presenting some form of CVD [4]. Although CVD remains the leading cause of death, in the recent years, several studies showed a positive trend for non-CVD deaths [5, 6] and a negative trend for CVD mortality in HD patients.

The aim of our multicenter study was to compare two cohorts of HD-treated patients (2012 and 2017) and to evidence trends and changes in mortality, comorbid conditions, prognosis, and causes of death after 5 years of continuous evolution of CKD management and treatment in Romania.

## Materials and methods

We included two cohorts of stable HD patients (901 from 2012 and 1396 from 2017) treated in the same 8 hemodialysis centers in Romania. Both cohorts had been followed up for 1 year. In all cases, HD therapy was performed using high flux, high surface dialyzers, three times/week (≥ 12 h/week). The assessment of therapy and follow-up of anemia and chronic kidney disease-mineral and bone disorder (CKD-MBD) were performed according to KDIGO guidelines [7, 8]. Personal data, history of disease, and HD therapy parameters were retrieved from patients’ dialysis files. Besides the clinical evaluation, the patients also underwent a cardiology assessment. A two-dimensional and M-mode continuous and Pulse Doppler echocardiography were performed in accordance with the recommendations of the European Association of Cardiovascular Imaging (EACI), between the second and third hour of the dialysis session, by the same operator for each cohort, using similar devices (avoid inter-observer differences). The operator assessed the left ventricular ejection fraction (LVEF) using the Simpson method, left ventricular hypertrophy and noted the presence and number of heart valve calcifications.

The 5-year survivors from 2012 cohort have been identified in 2017 and their data changes were assessed.

The Ethics Committee of each Dialysis Center approved the study and all patients signed a written informed consent to permit the investigators to use their personal data for scientific purposes. The study was performed in accordance with the Ethics Code of the World Medical Association.

### Statistical analysis

Data are presented as mean ± standard deviation (numerical variables with Gaussian distribution), median and interquartile range (numerical variables with non-Gaussian distributions), respectively, percentage from the sub-group total and number of individuals. Continuous variables distribution was tested for normality using Shapiro–Wilk test and for equality of variances using Levene’s test. Adjusted risk estimates for all-cause mortality were calculated using univariable and multivariable Cox regression. In this study, a *p* value of 0.05 was considered the threshold for statistical significance. Data were analyzed using SPSS v26 statistical software package (SPSS Inc, Chicago, IL, USA).

## Results

Two cohorts of ESKD patients, treated with HD in the same HD centers (901 patients from 2012 and 1396 from 2017) were analyzed. The baseline characteristics (Table 1) showed that the patients from 2017 were older (59.8 ± 13.1 vs 57.9 ± 13.3 years, *p* = 0.001) and had a longer time on dialysis therapy (5.8 ± 4.7 vs 4.7 ± 4.1 years, *p* < 0.001). The 2017 cohort had higher average serum creatinine (8.5 ± 2.4 vs 8.3 ± 2.3 mg/dl, *p* = 0.004) and urea (127 ± 34.6 vs 121.4 ± 38.9 mg/dl, *p* < 0.001) levels before dialysis and needed higher average ultrafiltration volume/dialysis (2467.8 ± 956.2 vs 2062.5 ± 1107.2 ml, *p* < 0.001). The average eKt/V, 1.4, as measured by ADIMEA, did not differ.

**Table 1:** Baseline data of the investigated cohorts

	2012 (*N* = 901)	2017 (*N* = 1396)	*p*
Age (A + SD)	57.9 (13.2)	59.8 (13.1))	**0.001**^**1**^
Gender (female)	381 (42.3%)	575 (41.2%)	0.633
Dialysis treatment (years) (A + SD)	4.7 (4.1)	5.8 (4.7)	** < 0.001**
Deaths (%)	60 (6.6%)	197 (14.1%)	** < 0.001**
Creatinine (mg/dl) (A + SD)	8.3 (2.3)	8.5 (2.4)	**0.004**^**1**^
Urea (mg/dl) (A + SD)	121.4 (38.9)	127.0 (34.6)	**0.032**
eKt/V (A + SD)	1.4 (0.3)	1.4 (0.4)	0.909
Dry weight (kg) (A + SD)	73.8 (29.0)	74.4 (17.4)	0.573^1^
Ultrafiltration volume (ml) (A + SD)	2062.5 (1107.2)	2467.8 (956.2)	** < 0.001**^**1**^
Dialyzer surface (A + SD)	2.1 (0.2)	2.1 (0.2)	0.675^1^
Qb (ml/min) (A + SD)	318.3 (47.0)	321.8 (44.3)	0.066^1^
Qd (ml/min) (A + SD)	673.5 (86.5)	580.9 (63.3)	** < 0.001**^**1**^
Vascular access (catheters) (%)	87 (9.7%)	255 (18.3)	** < 0.001**^**2**^
Hemoglobin (g/dl) (A + SD)	11.3 (1.3)	11.1 (1.4)	** < 0.012**^**1**^
Serum ferritin (ng/ml) (A + SD)	743.4 (483.1)	1025.2 (685.9)	** < 0.001**^**1**^
Transferrin saturation (%)(A + SD)	35 (16.5)	33.0 (14.5)	**0.002**^**1**^
Serum C-reactive protein (mg/dl) (A + SD)	7.9 (18.3)	1.2 (2.0)	** < 0.001**^**1**^
Serum albumin (g/dl) (A + SD)	4.0 (0.4)	4.1 (0.4)	** < 0.001**^**1**^
Iron sucrose (100 mg/vial) (vials/month) (A + SD)	2.0 (1.7)	1.2 (1.2)	** < 0.001**^**1**^
Darbepoetin alfa (micrograms/month) (A + SD)	67.2 (56.8)	68.1 (60.5)	0.723^1^
Serum calcium (mg/dl) (A + SD)	8.5 (0.7)	8.8 (0.7)	** < 0.001**^**1**^
Serum phosphate (mg/dl) (A + SD)	4.7 (1.4)	4.8 (1.5)	0.070^1^
Ca × P (mg^2^/dl^2^) (A + SD)	40 (12.1)	42.9 (13.4)	** < 0.001**^**1**^
Serum bicarbonate (mmol/l) (A + SD)	21.1 (4)	21.4 (2.8)	**0.018**^**1**^
iPTH (pg/ml) (A + SD)	551.5 (627.9)	528.1 (526.9)	0.336^1^
Hepatitis B (%)	46 (5.1%)	75 (5.4%)	0.854^2^
Hepatitis C (%)	199 (22.1%)	183 (13.1%)	** < 0.001**^**2**^
Parathyroidectomy (%)	50 (5.5%)	153 (11%)	** < 0.001**^**2**^
Peripheral vascular disease (%)	260 (28.9%)	365 (26.1%)	0.168^2^
Cerebrovascular disease (%)	183 (20.3%)	256 (18.3%)	0.263^2^
Diabetes mellitus (%)	188 (20.9%)	358 (25.6%)	**0.010**^**2**^
Heart rate disorders (EKG) (%)	151 (16.8%)	286 (20.5%)	**0.030**^**2**^
Myocardial infarction history (%)	102 (11.3%)	143 (10.2%)	0.455^2^
Ejection fraction (%) (A + SD)	58.2 (9.6)	56.8 (8.8)	** < 0.001**^**2**^
Heart valve calcifications (%) -1 valve	501 (55.6%)	1056 (75.6%)	** < 0.001**^**2**^
Heart valve calcifications (%) -2 Valves	61 (6.7%)	48 (3.4%)	**0.0003**
Left ventricular hypertrophy (%)	625 (69.4%)	1188 (85.1%)	< 0.001^2^

*A* average, *SD* standard deviation, *kg* kilograms, *ml* milliliters, *min* minute, *Qb* blood flow, *Qd* dialysis solution flow, *g* gram, *mmol* millimoles, *l* liters, *dl* deciliters, *ng* nanograms, *mg* milligrams, *EKG* electrocardiogram;

^1^*t* test

^2^Pearson’s Chi-squared test

Bolded numbers emphasize statistical significant results

In the 2017 cohort, the patients had lower average serum hemoglobin (*p* = 0.012), higher ferritin (*p* < 0.001), lower transferrin saturation (*p* = 0.002), lower C-reactive protein (*p* < 0.001), and higher albumin levels (*p* < 0.001). Regarding mineral bone disorders, the 2017 cohort presented higher average calcium levels (*p* < 0.001) and higher serum bicarbonate (*p* = 0.018) but phosphate and intact parathyroid hormone (iPTH) did not significantly differ. The prevalence of parathyroidectomy, as expected, was higher (*p* < 0.001).

Statistical differences were identified between HD specifications, medication, and vascular access. The 2017 cohort had lower average dialysis solution flow (Qd) (*p* < 0.001), patients were treated with lower iron sucrose doses (*p* < 0.001), and HD was performed more on catheters (18.3 vs. 9.7%, *p* < 0.001). The differences between 2017 and 2012 cohorts concerning comorbidities were: lower hepatitis C virus infection prevalence (*p* < 0.001), higher prevalence of diabetes mellitus (DM) as primary cause of ESKD (*p* = 0.01), higher prevalence of heart valve calcifications (*p* < 0.001), left ventricular hypertrophy (*p* < 0.001), heart rate disorders (electrocardiography proven) (*p* = 0.03) as well as lower average LVEF (*p* < 0.001) (Table 1).

Each cohort was followed up for 1 year. The 1-year mortality rate among stable dialysis patients was higher in the 2017 cohort (14.1 vs 6.6%, *p* < 0.001). In the 2012 cohort, the deceased patients were older (63.1 ± 12.3 vs. 57.9 ± 13.2 years *p* = 0.0031), had lower average Hb levels (10.3 ± 1.9 vs. 11.3 ± 1.3 g/dl, *p* < 0.0001), and higher average CRP levels (28.2 ± 31 vs. 7.9 ± 18.3 mg/dl, *p* < 0.0001) as compared with the average baseline values.. The deceased patients from the 2017 cohort were older (67.5 ± 10.6 vs. 59.8 ± 13.1 years, *p* < 0.0001), were treated with HD for a shorter period of time (4.6 ± 4.1 vs. 5.8 ± 4.7 years, *p* = 0.0007), had a lower average Hb level (9.9 ± 1.9 vs. 11.1 ± 1.4 g/dl, *p* < 0.0001), higher CRP levels (4.2 ± 5.7 vs. 1.2 ± 2.0 mg/dl, *p* < 0.0001), lower serum albumin levels (3.6 ± 0.6 vs. 4.1 ± 0.4 g/dl, *p* < 0.0001), and lower calcium levels (8.6 ± 0.8 vs 8.8 ± 0.7 mg/dl, *p* = 0.0002) as compared with the baseline data.

Concerning the causes of death, there were no statistical differences among the two cohorts. Cardiovascular disease was the leading cause of death (acute myocardial infarction, heart failure, arrhythmias, stroke, and sudden death), accounting for 50% of the patients from the 2012 cohort and 45.6% patients from the 2017 cohort. Sepsis remained the second most important cause of death (23.3% in 2012 and 20.8% in 2017). In the 2017 cohort, there was a downward trend in mortality from CVD with an increase in mortality caused by cancer without statistically significant differences (8.3–13.2%) (Table 2).

**Table 2:** Causes of death in the 1-year follow-up in 2012 and 2017

Causes of death	2012 *N* = 60 (%)	2017 *N* = 197 (%)	*p*
Acute myocardial infarction	5 (8.3)	20 (10.2)	0.677^1^
Hearth failure	8 (13.3)	17 (8.6)	0.282^1^
Arrythmias	2 (3.3)	10 (5.1)	0.575^1^
Sudden death	6 (10.0)	9 (4.6)	0.116^1^
Stroke	9 (15.0)	34 (17.3)	0.681^1^
Sepsis	14 (23.3)	41 (20.8)	0.677^1^
Cancer	5 (8.3)	26 (13.2)	0.311^1^
Hyperkalemia	2 (3.3)	5 (2.5)	0.740^1^
Withdraw from dialysis	0 (0.0)	3 (1.5)	0.336^1^
Others/unknown	9 (15.0)	32 (16.2)	0.818^1^

^1^*t* test

We analyzed several parameters that could modify mortality risk. Mortality risk in both cohorts was increased with: dialysis duration (OR = 1.179 CI 1.086–1.28) (*p* < 0.01), age (in both univariable and multivariate models) (OR = 1.05 CI 1.04–1.07, *p* < 0.001), the odds of death were 1.05 times higher for each 1-year increase in age, C-reactive protein (in multivariate but not univariate model) (OR = 1.02 CI 1.01–1.03, *p* < 0.001), the odds of death were 1.02 times higher for each one-unit increase in C-reactive protein level. For phosphate level (in multivariate but not univariate model), the odds of death are 1.19 times higher for each one-unit increase (CI 1.05–1.35, *p* = 0.007). For hemoglobin levels (in both univariable and multivariate model), the odds of death are 0.60 times lower (CI: 0.54–0.65, *p* < 0.001) for each one-unit increase. For albumin levels (in both univariable and multivariate models), the odds of death are 0.11 times lower for each one-unit increase (CI 0.08–0.15, *p* < 0.001) (Table 3).

**Table 3:** Univariate and multivariate analysis of factors associated with mortality in hemodialysis patients

Parameter	OR (univariate)	OR (multivariate)
Duration of dialysis	1.179 (1.086–1.28), *p* < 0.01	1.209 (1.09–1.31), *p* < 0.001
Age	1.05 (1.04–1.07), *p* < 0.001	1.06 (1.04–1.07), *p* < 0.001
CRP	1.02 (1.01–1.03), *p* < 0.001	0.96 (0.93–0.99), *p* = 0.012
Albumin	0.11 (0.08–0.15), *p* < 0.001	0.18 (0.12–0.27), *p* < 0.001
Po4	1.02 (0.92–1.12), *p* = 0.734	1.19 (1.05–1.35), *p* = 0.007
Hemoglobin	0.60 (0.54–0.65), *p* < 0.001	0.65 (0.58–0.73), *p* < 0.001
iPTH	1.00 (1.00–1.00), *p* = 0.503	1.00 (1.00–1.00), *p* = 0.090
Ferritin	1.00 (1.00–1.00), *p* = 0.085	1.00 (1.00–1.00), *p* = 0.079

*CRP* C-reactive protein, *PO4* seric phosphate, *iPTH* intact parathormone, *OR* odds ratio

In 2017, we identified 566 survivors out of the 901 stable HD patients from 2012 cohort. The 5-year survival rate was 62.8% with the average mortality rate of 7.44%/year. All the patients that changed HD center or underwent renal transplant in the 5-year period were excluded. The patients’ characteristics were analyzed to determine the changes after 5 years of HD therapy. *t* test and Chi-square tests showed that there are several significant statistical differences concerning patient’s medical data in the 5-year survival time, 56 (9.8%) patients died in 2017. As expected, the prevalence of catheter increased in HD (from 6.71 to 13.95%, *p* < 0.001). In 2017, the patients presented (before the dialysis session) higher average serum creatinine (*p* < 0.004), similar average dry weight and serum bicarbonate levels, increased average albumin (*p* < 0.001), and lower C-reactive protein (*p* < 0.001) levels, suggesting a good nutrition status and decreasing inflammation. Similar hemoglobin, transferrin saturation levels and similar average epoetin doses but with higher average ferritin levels (*p* < 0.0001) could be related to higher on average monthly doses of intravenous iron therapy (*p* < 0.001). The higher on average serum calcium (*p* < 0.001), higher calcium-phosphate product (*p* = 0.004), and higher PTH levels (*p* = 0.022) with similar phosphate levels was a result of excess calcium-based phosphate binders and/or vitamin D (and/or vitamin D analogs). One should also mention the higher number of patients treated with parathyroidectomy (*p* < 0.001) (Table 4). In time, eKt/V remained similar although the filter surfaces and Qb increased.

**Table 4:** Characteristics of 5-year HD therapy survivors from 2012 to 2017

	2012 (*N* = 566)	2017 (*N* = 566)	*p*
Age (A + SD)	55.6 (12.8)	59.8 (12.8)	** < 0.001**^**3**^
Creatinine (mg/dl) (A + SD)	8.6 (2.3)	9.0 (2.3)	** < 0.004**^**3**^
Urea (mg/dl) (A + SD)	125.1 (35.8)	130.0 (34.6)	** = 0.024**^**3**^
eKt/V (A + SD)	1.4 (0.3)	1.4 (0.2)	= 0.528^3^
Dry weight (kg) (A + SD)	73.6 (16.8)	72.6 (17.0)	= 0.356^3^
Ultrafiltration volume (ml) (A + SD)	2102.5 (1118.0)	2497.7 (930.5)	** < 0.001**^**3**^
Dialyzer surface (A + SD)	2.1 (0.2)	2.2 (0.2)	** < 0.001**^**3**^
Qb (ml/min) (A + SD)	325.0 (46.8)	334.0 (42.7)	** = 0.001**^**3**^
Qd (ml/min) (A + SD)	680.6 (86.7)	600.0 (68.8)	** < 0.001**^**3**^
Vascular access (catheters) (%)	38 (6.71%)	79 (13.95%)	** < 0.001**^**2**^
Hemoglobin (g/dl) (A + SD)	11.3 (1.2)	11.2 (1.4)	= 0.184^3^
Serum ferritin (ng/ml) (A + SD)	756.2 (502.7)	1202.6 (819.6)	** < 0.001**^**3**^
Transferrin saturation (%) (A + SD)	35.7 (16.9)	35.0 (15.4)	= 0.471^3^
Serum C-reactive protein (mg/dl) (A + SD)	6.2 (15.3)	1.1 (1.9)	** < 0.001**^**3**^
Serum albumin (g/dl) (A + SD)	4.1 (0.4)	4.2 (0.4)	** < 0.001**^**3**^
Iron sucrose (100 mg/vial) (vials/month) (A + SD)	1.9 (1.6)	1.1 (0.9)	** < 0.001**^**3**^
Darbepoetin alfa (micrograms/month) (A + SD)	64.3 (56.3)	64.9 (59.8)	= 0.869^3^
Serum calcium (mg/dl) (A + SD)	8.5 (0.7)	8.9 (0.8)	** < 0.001**^**3**^
Serum phosphate (mg/dl) (A + SD)	4.8 (1.4)	4.8 (1.5)	= 0.757^3^
Ca x Pi (mg(2)/dl(2)) (A + SD)	40.8 (12.4)	43.1 (14.5)	** = 0.004**^**3**^
Serum bicarbonate mmol/l (A + SD)	21.0 (3.9)	21.4 (2.8)	= 0.098^3^
iPTH (pg/ml) (A + SD)	555.1 (592.5)	640.5 (631.5)	** = 0.022**^**3**^
Parathyroidectomy (%)	34 (6%)	106 (18.72%)	< 0.001^2^
Hepatitis B (%)	25 (4.41%)	28 (4.94%)	= 0.67^2^
Hepatitis C (%)	117 (20.67%)	124 (21.9%)	= 0.61^2^
Coronary artery disease (%)	354 (60.9%)	394 (69.6%)	** = 0.004**^**2**^
Peripheral vascular disease (%)	123 (21.7%)	157 (27.7%)	** = 0.023**^**2**^
Cerebrovascular disease (%)	87 (15.3%)	111 (19.6%)	= 0.075^2^
Diabetes mellitus (%)	95 (16.7%)	114 (20.1%)	= 0.173^2^
Heart rate disorders (EKG) (%)	77 (13.6%)	130 (22.9%)	** < 0.001**^**2**^
History of myocardial infarction (%)	48 (8.4%)	70 (12.3%)	** = 0.037**^**2**^
Ejection fraction % (A + SD)	59.2 (9.4)	57.5 (8.8)	** = 0.003**^**3**^
Heart valve calcifications (%) -1 valve	290 (51.2%)	434 (76.6%)	** < 0.001**^**2**^
Heart valve calcifications (%) -2 valves	37 (6.5%)	39 (6.8%)	
Left ventricular hypertrophy (%)	371 (65.5%)	508 (89.7%)	** < 0.001**^**2**^

*A* average, *SD* standard deviation, *kg* kilograms, *ml* milliliters, *min* minute, *Qb* blood flow, *Qd* dialysis solution flow, *g* gram, *mmol* millimoles, *l* liters, *dl* deciliters, *ng* nanograms, *mg* milligrams, *EKG* electrocardiogram

^1^*t* test

^2^Pearson's Chi-squared test

Bolded numbers emphasize statistical significant results

Also, as expected, in 2017, the patients presented more comorbid conditions compared to 2012: more coronary artery disease (*p* = 0.004), more peripheral vascular disease (*p* = 0.023), more heart rate disorders, (p < 0.001), more history of myocardial infarction (*p* = 0.037), more LVH (*p* < 0.001), more valvular calcifications (*p* < 0.001), and lower average ejection fraction (*p* = 0.003). (Table 4).

## Discussion

ESKD patients treated with HD are at high risk for CVD with increased mortality under dynamic changes induced by the cumulative risk factors of CKD, increased pre-dialysis and dialysis survival as a result of better medical care (i.e., lowering CKD progression, preventing and treating CVD, and advanced CKD complications) and personalized HD therapy [9, 10]. Under these conditions, assessing the changes in the HD population, the trends in evolution, mortality and causes of death represent an important tool for future medical strategies [11, 12]. Therefore, in our multicenter study, we compared the baseline data of HD patients (at entry in the study) in two cohorts, 2012 and 2017, performing HD therapy in the same eight centers from Romania. In 2012, according to the Romanian Renal Registry (RRR), 9551 patients were performing HD therapy for ESKD from which 9.4% (901) were represented by our cohort [13]. The annual increase of HD patients was 7.8%/year reported by the same registry. In 2017, the cohort from the same 8 centers was represented by 1396 patients. RRR reported that 13,362 patients were on HD in 2017, with 10.4% (1396) representing our second cohort. The average annual increase of patients in the eight centers was 10.9% (being higher than the national average). One should evidence the fact that the prevalence of HD patients decreased in Romania in 2020 with 1.4% most probably related to the Corona virus pandemic [14].

The baseline data of the two cohorts (2012 and 2017) had many differences. In 2017, the patients were significantly older (59.8 vs 57.9 years, *p* = 0.001) and were, on average, on dialysis treatment for a longer time (5.8 vs 4.7 years, *p* < 0.001). In our cohorts, the mean age of HD patients increased, in 2017 being close to the mean age reported by ERA-EDTA (60.7 years). One should mention the fact that 566 (40.5%) patients of the 2017 cohort were 5-year survivals from 2012. Therefore, age differences, HD duration time, and mortality are influenced by these high number of 5-year survivals. There were, however, important differences between European countries concerning mean age of prevalent HD patients (Albania 49.5 years, Ukraine 50, Scotland 56.8, Spain 59.5, Denmark 59, Portugal 67.9) [15]. In Europe (and in our cohorts also), there is a trend for increasing age in prevalent HD patients (mean age being 61.8 years in the 2019 ERA-EDTA registry). Age and duration of HD therapy have been associated to higher risk of mortality in these patients. Though a decrease in mortality was registered among USRDS reporting patients between 2010 and 2019, the 2020 mortality increased in all age groups. The largest absolute increase was evidenced in the older patient’s group [17, 18].

Average pre-dialysis serum creatinine and urea were significantly higher in the 2017 cohort (*p* < 0.004 and *p* < 0.032, respectively) but urea to creatinine ratio (UCR) did not differ. C-reactive protein (CRP) was lower (*p* < 0.001) and serum albumin was higher (*p* < 0.001) in the same cohort suggesting a lower inflammation and improved nutrition status but average dry weight did not significantly differ. Higher pre-dialysis serum creatinine and UCR were associated with increased risk of death in HD patients [19, 20] more or less related to malnutrition inflammation complex syndrome. In our 2017 cohort, this relation could not be evidenced since average CRP values and average dry body weight were not modified as expected. Most probably our results could be related to a better quality of the diet.

Lower average hemoglobin and transferrin saturation values and higher ferritin levels in context of near to normal average CRP (12 mg/l) were the characteristics of the 2017 cohort (as compared to 2012). It seems that the average higher ferritin levels are the result of higher intravenous iron therapy similar to that proposed by the PIVOTAL study [21]. Higher ferritin levels seem to be associated with increased mortality risk in HD patients but when corrected to malnutrition and inflammation, the risk seems to be attenuated [22]. Nevertheless, the average intravenous iron dose was adapted to ferritin levels and was reduced significantly in the 2017 cohort (from 200 mg/month in 2012 to 120 mg/month in 2017, *p* < 0.001).

In the 2017 cohort, the average serum calcium and CaxP product were significantly higher (8.8 ± 0.7 vs 8.5 ± 0.7 mg/dl *p* < 0.001 and 42.9 ± 13.4 vs 40 ± 12.1 mg^2^/dl^2^, *p* < 0.001, respectively) but average phosphate, iPTH, and vitamin D levels did not differ. These results could be related to higher need for phosphate binders and excessive use of calcium-based ones. The influence of calcium-based vs. non-calcium-based phosphate binders on the risk of death in HD patients is a still debated issue. Papers supporting decreased risk of all-cause mortality in HD patients using non-calcium-based phosphate binders vs. calcium-based ones [23, 24] to no effect or differences [25] between treatments have been published in the recent years. In our opinion, the conclusions of a 2018 Cochrane meta-analysis deserve to be mentioned [26]. Though Sevelamer may lower the risk of death compared to calcium-based binders, the authors find no benefits for any phosphate binder concerning cardiovascular death, myocardial infarction, coronary artery calcifications or stroke. In our cohorts, the differences mentioned above between the two cohorts did not influence the risk of death.

The dialysis adequacy in the 2017 cohort (as compared to 2012) was similar. The average targeted eKt/V of 1.4 was reached in both cohorts. Both cohorts were treated with high flux dialyzers,  ≥ 12 h/week. Higher ultrafiltration was needed in 2017 (on average 8.29 vs. 6.98 ml/kg/h) and the average dialysis fluid flux (Qd) was lower. Prolonged fluid overload as well as higher ultrafiltration volumes have been related to increased mortality in HD patients [27–29], though no clear cutoff levels for optimal ultrafiltration are documented until now. As we will discuss further, higher ultrafiltration rates in the 2017 cohort did not increase mortality risk.

The baseline data from 2017 reveal a higher prevalence of some comorbid conditions and complications. The prevalence of DM as primary condition for ESKD increased (25.6 vs. 20.9%, *p* = 0.010). It is an expected result since prevalence of DM is increasing worldwide and ESRD related to DM, also [30, 31], though the incidence rates for DM-related ESKD are significantly lower in Europe as compared to US. The mortality of DM-related ESKD patients seems to be also significantly higher as compared to the no DM ESKD patients and this may influence the survival estimates in HD patients in the future [32–34]. The prevalence of hepatitis B virus infection did not change in the two cohorts being around 5%, but the hepatitis C virus infection decreased as a result of a nationwide prevention program applied since 2000 and from 2015 a nationwide treatment program with the novel direct oral antivirals which included HD patients also. In our two cohorts hepatitis virus infection did not increase mortality risk.

The prevalence of cerebrovascular disease, peripheral vascular disease, and history of myocardial infarction was not higher among the 2017 cohort HD patients. Nevertheless, patients presented higher prevalence of left ventricular hypertrophy, lower average ejection fraction, and higher prevalence of heart valve calcification (one valve/two valves) (see Table 1). The causes seem to be complex: higher age of the HD-treated patients, on average longer period on HD therapy, increased prevalence of DM 2 related ESKD. One should also evidence the fact that 566 HD patients from the 2012 cohort (62.8%) survived and could be identified in the 2017 cohort (representing 40.5% of the 2017 patients) (Table 3.). The 5-year unadjusted survival rate was very high if compared with the data from USA, Europe, and Japan Registries for patients initiating HD between 2004 and 2008 (39%, 41%, and 60%, respectively) [35, 36]. The possible explanations should be that the 5-year survivors from 2012 were significantly younger at the inclusion in our study (around 55 years), they performed HD using mainly Cimino type shunts (only 6.7% on catheters), had higher Hb levels, lower prevalence of DM, lower prevalence of cardiovascular complications, and higher average LVEF (Table 3). Similar results were presented by the ANZDATA for non-indigenous patients initiating HD between 2009 and 2018 for the age groups between 45 and 64 [37]. During the 5-year survival, as expected, all those characteristics became significantly worse, influencing the baseline data of the 2017 cohort and the mortality also (the 1-year mortality being 6.6% in 2012 vs. 14.1% in 2017). On the other hand, in the last 2 years, increasing mortality was reported by the USRDS also [18].

Concerning main causes of death, we found no significant differences between the two cohorts. Cardiovascular disease remained the main cause of death. Though a decreasing trend was registered (45.6% in 2017 of all causes of death vs. 50% in 2012), the difference did not reach statistical significance. Decreasing trends in cardiovascular mortality among HD patients have been reported by many national and regional registries. However, cardiovascular disease remains the main cause of death in HD-treated ESRD patients (Australia 30.6%, New Zeeland 36.2%, USA 2020 51.5%, Europe 39%) [16, 18, 37]. The 2020 Romanian Renal Registry evidenced a cardiovascular cause mortality of 53% in Romanian HD patients [14]. In our cohorts, sepsis was the second cause of death with a decreasing prevalence from 2012 to 2017 (23.3–20.8%—similar with the Romanian Renal Registry in 2018 i.e., 19%), still, remaining higher than reported by other registries (Australia 8.9%, USA 9.1%, New Zeeland 10.8%, and 16.2% Europe 2015) [14, 18, 37, 38]. Cancer-related deaths in our cohort had an increasing trend, from 8.3% in 2012 to 13.2% in 2017. Cancer-related deaths varied across the national/regional registries also, being 1.4% in New Zeeland, 2.3% in the USA, 4. 4% in Australia, and 7.8% in Europe [18, 37, 38]. Our data are higher than those reported by the Romanian Renal Registry (4%). It is evident that mortality and causes of death vary across countries/regions depending on socioeconomic status, renal replacement therapy (RRT) strategies, RRT practice patterns, disparities in access to treatment, detection of CKD and pre-dialysis care and treatment of CKD, and so on [36]. Even coding and reporting collected data to the registries may influence results as it was recently evidenced by comparing data published by USRDS and the Kaiser Permanente integrated health care system from California [39].

Multiple risk factors influence mortality in HD patients. In a 2017 meta-analysis (23 studies included), risk for all-cause mortality in HD patients was increased by age, presence of DM, previous CVD, higher CRP levels, higher levels of ferritin, higher levels of HbA1c, TnT and BNP, while higher BMI, hemoglobin, albumin, TIBC, ApoA2 and ApoA3 levels were associated with lower risk. Cardiovascular mortality risk was increased by age, gender (women vs. men), DM, previous CVD, HD duration, higher levels of ferritin, HDL, and HbA1c. Higher albumin, TIBC, ApoA2 levels turned out to be associated with lower risk. Worth to be mentioned the fact that mortality risk factors differ in Western and Eastern countries [40]. Differences concerning mortality risk exist between countries, HD centers, geographic areas, mainly dependent of group of investigated patients and collected data. For example, in Japan (2007), mortality risk was increased by high pulse pressure, presence of cerebrovascular disease, lower serum creatinine levels and low **e**Kt/V [41]; in Spain (2021) in a community HD center, risk of mortality was associated to older age, acute deterioration of chronic kidney disease, use of catheters and hypoalbuminemia [42]. In our two cohorts, the risk of mortality increased related to dialysis duration, age, high phosphate and C-reactive protein levels. Higher hemoglobin and albumin levels turned out to be protective.

## Conclusion

The prevalence of ESRD patients needing HD therapy is increasing in our East-European population. More and more HD is performed at older ages and in higher number of DM patients. The 5-year survival is high in our cohorts (more than 62% of the cases) based mainly on good-quality HD therapy and care. There are decreasing trends in cardiovascular mortality and sepsis-related deaths and increasing cancer-related mortality. Nevertheless, cardiovascular disease remains the main causes of death. If compared to other studies and annual data reports of regional and country registries, results are widely differing related to socioeconomic status, quality of care and therapy, and different reporting systems. Addressing modifiable risk of mortality and unifying reporting systems should decrease mortality in HD-treated ESRD patients and would permit developing regional therapeutic strategies to provide a better survival of HD patients.
